# Percutaneous Gastrostomy Tube Placement under Quadratus Lumborum Block: A Case Report

**DOI:** 10.3390/medicina59122106

**Published:** 2023-11-30

**Authors:** Gundega Ose, Irina Evansa, Nikita Ivanovs, Natalija Zlobina, Indulis Vanags, Olegs Sabelnikovs

**Affiliations:** 1 Faculty of Medicine, Riga Stradins University, 1007 Riga, Latvia; 2 Anesthesiology, Intensive Care and Pain Department, Riga 1st Hospital, 1001 Riga, Latvia; 3 Department of Anesthesiology and Intensive Care, Riga Stradins University, 1007 Riga, Latvia; 4 Clinic of Anesthesiology and Reanimatology, Paul Stradins Clinical University Hospital, 1002 Riga, Latvia

**Keywords:** quadratus lumborum block, anesthesia, polymorbidity, gastrostomy tube

## Abstract

The quadratus lumborum block is a technique that is not widely applied in abdominal surgery. The influence of the mode of anesthesia on the outcome of polymorbid patients is a controversial issue in the medical literature. We report a case in which we performed a quadratus lumborum block type 2 on a woman who was admitted to Riga’s 1st hospital in need of gastrostomy, due to difficulty swallowing solid foods and liquids caused by hypopharynx carcinoma. On account of the patient’s difficult airway, general anesthesia was deemed unsafe for the patient, with a risk of patient death. Percutaneous gastrostomy tube placement under a quadratus lumborum block type 2 was performed successfully.

## 1. Introduction

The quadratus lumborum block is a block of the posterior abdominal wall, an “interfascial plane block”, performed only under ultrasound guidance. It was first described in 2007 by Dr Rafael Blanco as part of the TAP (transversus abdominis plane) block, but in 2016 it was recognized as a separate block type [1,2]. The QL block is divided into three types—type 1, type 2, and type 3. QLB type 1 target structures are located between the transversus abdominis aponeurosis at the lateral border of the quadratus lumborum muscle and the QLB type 3, also called “transmuscular QLB” target structure is located in the plane between the quadratus lumborum muscle and the psoas muscle, providing analgesia in the lower abdomen. In this case, the type 2 block, also called the posterior QL bock, was used due to its ability to provide adequate analgesia in the region of surgery. The target structure for the quadratus lumborum block 2 is the posterior side of the quadratus lumborum muscle (QLM)—between the QLM and the medial lamina of thoracolumbar fascia which separates the QLM from the latissimus dorsi muscle and erector spinae muscles. This is located laterally from the internal oblique muscle aponeurosis attachment to the so-called lumbar interfascial triangle [2,3]. The mechanism of action of QL blocks is still uncertain. The likely explanation is that the local anesthetic may spread through the diaphragm to reach the paravertebral space via the endothoracic pathway. This would result in the blockade of the cutaneous nerves of the abdominal wall and lower thoracic sympathetic trunk [4]. All block types are made to be used as an analgesia for the abdominal wall, but the QLBT2 is deemed as the safest option.

In light of everchanging tendencies in medicine as a whole and the rising popularity of regional anesthesia, general anesthesia is becoming the less desired type of anesthesia for polymorbid patients. In this case, we describe the application of a QLB in a 59-year-old woman who was admitted to Riga’s 1st hospital and underwent percutaneous gastrostomy tube (PGT) placement due to malnutrition caused by her inability to swallow solid foods and liquids due to hypopharynx carcinoma.

## 2. Case Presentation

**Figure 1:**
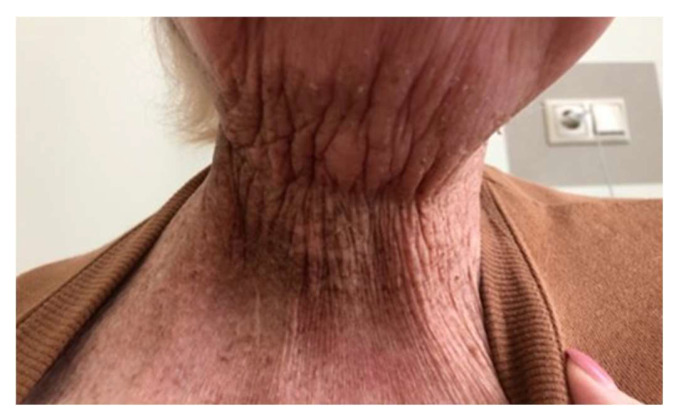
The hypopharynx carcinoma’s effect on patients’ skin and neck.

A 59-year-old woman with a BMI 16.8, suffering from hypopharynx carcinoma in the epiglottic area, T2N0M0 St.II., and severe malnutrition due to difficulties swallowing solid foods and liquids, was admitted to Riga’s 1st hospital for percutaneous endoscopic fibrogastroscopy (Figure 1).

**Figure 2:**
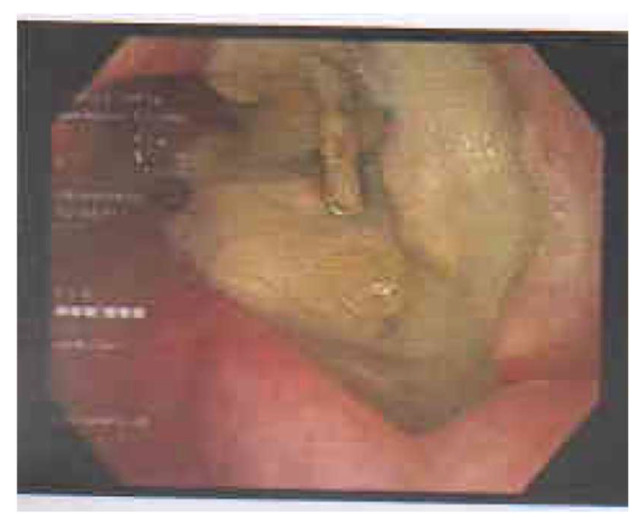
The outgrowth and necrotic tissue seen during endoscopy.

The patient was feeding through a nasogastric tube upon admission to the hospital. During an endoscopic examination, an outgrowth with necrotic tissue was seen (Figure 2).

The tumour was fully blocking the entrance to the oesophagus and all that was left was a small opening into the trachea. Upon further examination, metastasis in the colli dextra lymph nodes was found. In a previously performed magnetic resonance imaging study, the patient’s condition was defined as status after combined therapy of carcinoma of the right parapharyngeal region, extirpation of the sternocleidomastoid muscle dextra and extirpation of the internal jugular vein.

**Figure 3:**
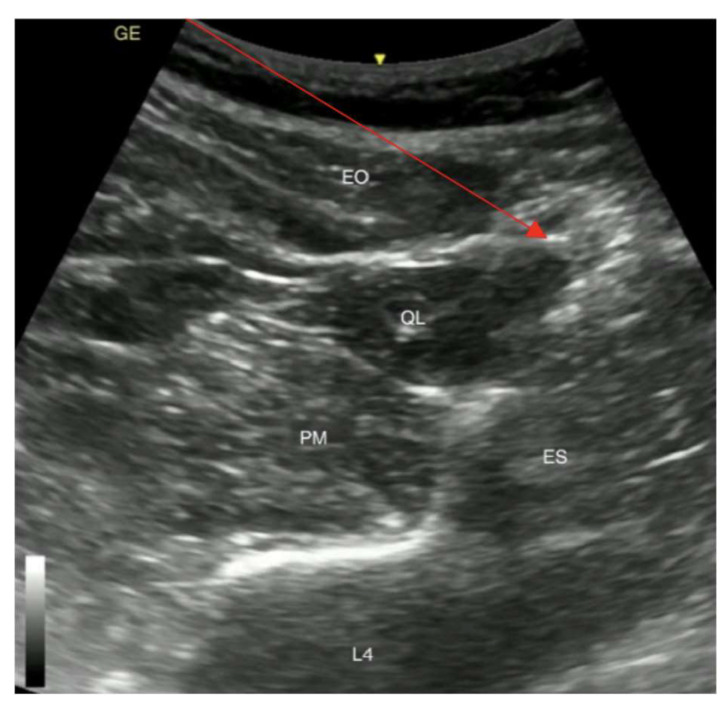
Posterior QLB (type 2). EO, external oblique muscle; QL, quadratus lumborum muscle; PM, psoas major muscle; ES, erectores spinae muscle; L4, fourth lumbar vertebra; Red arrow shows the needle path for posterior QLB. The yellow arrow shows patient’s skin.

The procedure was stopped because of the patient’s airway obstruction caused by the hypopharynx carcinoma, followed by a desaturation of SpO_2_ to 23% when 50 mg propofol was administered (1.2 mg/kg intravenous propofol). Following these dramatic complications, the patient was given intravenous Sol. Dexoni 8 mg and O_2_ lung ventilation with an Ambu bag was performed. The patient’s oxygen saturation returned to 86–92% after 6 min. As her oxygen saturation at rest was within the normal range, there were no indications for tracheostomy. After experiencing complications associated with anaesthetics and considering the possible risk with patient intubation and further desaturation, the decision for the placement of a gastrostomy tube was made in the operating room and the anaesthesia of choice was the quadratus lumborum block type 2 (QLB2) (Figure 3).

The left side QLB2 was performed under ultrasonographic control with a curvilinear probe with a 21 G 100 mm plexus needle and 20 mL of 0.375% ropivacaine was injected. The patient had pain and discomfort at the start of the surgery, mainly caused by the pre-existing condition, hypopharynx carcinoma. In addition, after the complications associated with the anaesthetics, the patient became nervous and emotional and the decision to administer fentanyl and ketamine to the patient was made, assuming the QLB had not yet reached its peak effect. Intraoperatively the patient received a total of 200 mcg of fentanyl by fractions of 50 mcg and a ketamine total dose of 50 mg. No local infiltration of skin or other tissues was performed by the surgeon. The length of the surgery was 40 min. Analgesia consisted solely of 500 mg of paracetamol 30 min before the end of the procedure.

**Figure 4:**
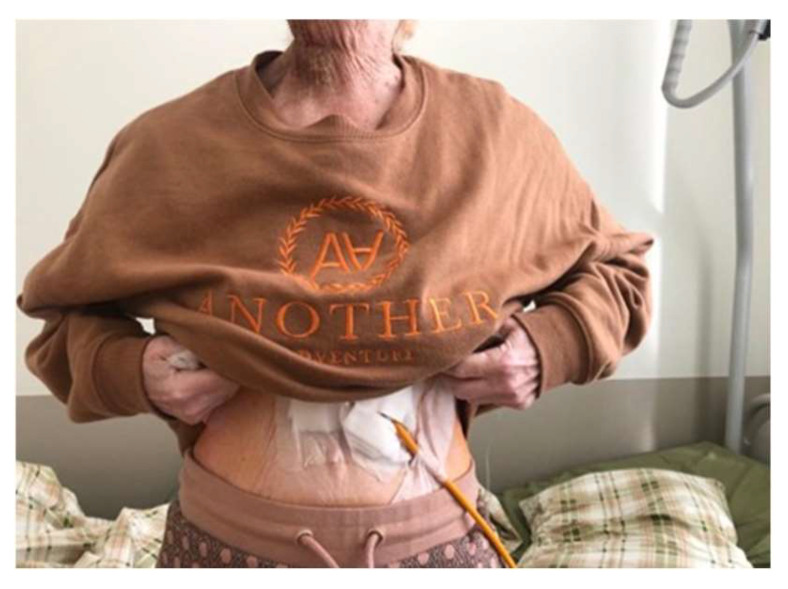
Patient after the surgery.

The patient was awake during the tube placement, intraoperatively she reported the sensation of pain using a numeric rating scale (NRS) from 1 to 10. The patient reported no intraoperative pain. The procedure was successful, and the patient was given a functional and sufficient way of feeding herself, preventing further malnutrition (Figure 4).

The patient reported no pain during her stay at the PACU; mild pain was mentioned in the ward 8 h after the block. Postoperatively the patient received multimodal analgesia with NSAIDs and acetaminophen. The first injection of Sol. Promedoli 2% 1.0 mL (Sol. Trimeperidini 20 mg), which is the equivalent of 5 mg morphine, was administered 10 h after the surgery when the patient reported pain of 7 (NRS).

The patient was discharged from the hospital the day after the surgery. No further complications were reported in relation to the gastrostomy.

## 3. Discussion

A quadratus lumborum block traditionally provides sufficient sole postoperative analgesia for a large number of surgical interventions, such as gynecological laparoscopic procedures or abdominal surgery [5,6]. QLB2 provides large sensory inhibition of T7-L1, and as the human dermatomes map out, the use of QLB2 in upper abdomen surgery becomes the logical step in polymorbid patients [6]. This case provides us with an insight into the future of surgery and the drift away from general anesthesia.

Regional anesthesia is also a way to prevent complications that may arise if the patient is undergoing general anesthesia. The most common complications include nausea, dizziness, and incision pain [7]. For a patient in a fragile state, like described in this case, these complications can be severe and significantly increase time spent in hospital, which can have negative consequences for the mental state of the patient and time needed for recovery.

In the presented case, the patient suffered from chronic pain caused by a tumor in the hypopharynx, so multimodal analgesia was the approach that was taken. The quadratus lumborum block successfully covered the post-operative pain in her upper abdomen.

There is a need for a bigger discussion on the variety of QLB block uses, as there is a wide range of contraindications for general anesthesia and a high risk of using it. The QLB2 block can provide sufficient analgesia in the upper abdomen surgeries for patients, reducing the need for epidural analgesia, decreasing the risk of infection, or providing a safer alternative in already septic patients [8].

As in the case presented in this article, the usual way of administering general anesthesia can sometimes result in patient death or unwanted complications. But doctors still have to be wary of the possible contraindications of the quadratus lumborum block. As with every invasive procedure, the possibility of an allergic reaction to medication or local anesthetics is present. There is currently no evidence for the stratification of the risk of bleeding based on quadratus lumborum approaches [4].

In a prospective study in 2016, by Murouchi T. et al., it was shown that the QL block, performed as a single injection of 20 mL 0.375% ropivacaine, could spread to T7-T12 and last for almost 24 h [9]. That means, that the quadratus lumborum block can be used to prevent both visceral and somatic pain in the upper abdomen.

The postoperative administration of pain medication and pain management in hospitals is an ongoing discussion. The common consensus worldwide is in favor of limiting opioid usage to the lowest possible dose. Mostly in postoperative care, opioids are administered based on the need expressed by the patient, which can sometimes be soon after waking up from anesthesia [10]. Our patient received the first injection of Sol. Promedoli 2% 1.0 mL 10 h after surgery.

The duration of analgesia can also play a key role in reducing not only immediate post-operative pain but can also be a tool in the treatment chronic neuropathic pain in the abdominal wall caused by previous surgical interventions [11].

In a case report by Kadam VR. in 2013, the use of QL block was shown to be a successful type of analgesia for laparotomy for duodenal tumor excision [12]. This further supports the idea of QL blocks being a tool for polymorbid patients in need of major abdominal surgery.

In the case of the percutaneous gastrostomy, the QLB2 was a success, as the placement of the tube is located around the 8/9 human dermatome, which is on the upper side of the QLB2 target location. In a case report made in 2019, by Yavuz G. et al., quadratus lumborum block can be safely used to perform such surgeries as right-sided nephrectomy and cholecystectomy, where the surgical incisions are performed higher up [13].

Not only does QLB2 provide good perioperative analgesia, but it has also been shown to reduce opioid consumption, as presented in this case. This shows that regional anesthesia can enhance postoperative recovery [3,14].

## 4. Conclusions

The influence of the mode of anesthesia on the outcome of polymorbid patients is a controversial issue in the medical literature. In light of an aging society, a conclusive answer to this question is of growing importance. If general anesthesia can lead to severe complications and even the death of the patient, regional anesthesia might be chosen as an alternative. This case presents that the quadratus lumborum block type 2 is a safe alternative to general anesthesia in percutaneous gastrostomy tube placement. It also sheds light on the benefits and success of regional anesthesia and the future possibilities of decreasing the risk of undergoing major surgery in polymorbid patients.

## Data Availability

Data is contained within the article.
